# Osteopontin‐induced lncRNA HOTAIR expression is involved in osteoarthritis by regulating cell proliferation

**DOI:** 10.1186/s12877-020-01993-y

**Published:** 2021-01-14

**Authors:** Quanzhi Liang, Ailijiang Asila, Yingjie Deng, Jun Liao, Zhenfeng Liu, Rui Fang

**Affiliations:** 1 Department of Orthopedics, Chinese Medicine Hospital of Xinjiang Uygur Autonomous Region, No. 116, Huanghe Road, Shayibake District, 830000 Urumqi City, Xinjiang Province People’s Republic of China; 2 Department of Rehabilitation, the Ninth People’s Hospital of Wuxi, 214000 Wuxi City, Jiangsu Province People’s Republic of China

**Keywords:** Osteopontin, HOTAIR, Osteoarthritis, Chondrocyte, Proliferation

## Abstract

**Background:**

Osteopontin plays critical roles in osteoarthritis (OA) by regulating the functions of osteoclasts. It is known that osteopontin can induce the expression of lncRNA HOX transcript antisense RNA (HOTAIR), indicating the involvement of HOTAIR in OA. This study was carried out to investigate the role of HOTAIR in OA.

**Methods:**

Synovial fluid was extracted from both OA patients (*n* = 58) and healthy controls (*n* = 58). Expression of osteopontin and HOTAIR in synovial fluid was determined by RT-qPCR. Osteopontin was used to treat chondrocytes at dosages of 0, 1, 5 and 10 µg/ml, followed by measurement of HOTAIR expression by RT-qPCR. The role of osteopontin and HOTAIR overexpression, as well as HOTAIR knockdown in regulating the proliferation of chondrocytes was analyzed by cck-8 assay.

**Results:**

HOTAIR was upregulated in OA. A positive correlation between HOTAIR and osteopontin was observed. In the primary chondrocytes, osteopontin treatment increased HOTAIR expression, while HOTAIR overexpression and knockdown failed to significantly affect osteopontin expression. In addition, osteopontin and HOTAIR overexpression increased chondrocyte proliferation, while HOTAIRE knockdown decreased chondrocyte proliferation. In addition, HOTAIR knockdown reduced the effects of osteopontin treatment on cell proliferation.

**Conclusions:**

Osteopontin-induced HOTAIR expression is involved in osteoarthritis by regulating cell proliferation.

## Background

Osteoarthritis (OA) is the most frequently diagnosed arthritis among middle-age to elderly people. OA occurs during the breaks of cartilage that protects or cushions the ends of bones [1, 2]. Aging is the most common cause of primary osteoarthritis. With the increased use of joints, damaged cartilages will be lead to swelling and joint pain [3]. It is estimated that more than 10% of males and 13% of females aged over 60 years are suffering from OA [4]. At present, there is no cure for OA, and medications mainly aim to relieve osteoarthritis symptoms, such as primarily pain [5]. Therefore, novel and more effective therapeutic approaches are still needed. However, molecular pathogenesis of OA remains hardly known, therefore the development of treatment strategies is limited [6, 7].

It has been well established that the initiation and progression of OA require the involvement of multiple molecular players [8, 9]. For instance, osteopontin, also known as Secreted Phosphoprotein 1 (SPP1), is a multifunctional phosphoprotein that is secreted by multiple cells types, such as chondrocytes and osteoblasts, is upregulated in OA and induces the production of matrix metalloproteinase 13 to promote the degradation of type II collagen, which is the major component of cartilage matrix [10, 11]. Therefore, osteopontin is considered as a target for the treatment of OA [10, 11]. Besides that, long non-coding RNAs (lncRNAs) also play crucial roles in OA by regulating OA-related gene expression [12]. In a recent study, osteopontin was reported to regulate lncRNA HOX transcript antisense RNA (HOTAIR) expression in cancer cells [13], suggesting the potential involvement of the interaction between osteopontin and HOTAIR in cancer. It is known that HOTAIR is overexpressed in OA, suppresses WIF-1 expression and activates Wnt pathway to promote OA progression [14]. This study was therefore carried out to explore the potential interaction between osteopontin and HOTAIR in OA.

## Methods

### Research subjects

From May 2018 to June 2019, 58 OA patients (23 males and 35 females, 52 to 70 years, 61.2 ± 5.7 years) and 58 healthy controls (23 males and 35 females, 52 to 70 years, 61.3 ± 5.9 years) were enrolled at Chinese Medicine Hospital of Xinjiang Uygur Autonomous Region. This study was approved by the Research Ethics Committee of Chinese Medicine Hospital of Xinjiang Uygur Autonomous Region and was in line with the Helsinki Declaration. Inclusion criteria: (1) newly diagnosed OA patients; (2) patients willing to donate synovial fluid. Exclusion criteria: (1) patients complicated with other clinical disorders; (2) recurrent OA; (3) initiated therapy within 3 months prior to admission. The diagnosis of OA was performed by both X-ray and joint fluid analysis. All healthy controls received systemic physiological exam at aforementioned hospital, and all physiological functions were normal. Written informed consent was provided by all patients and controls.

### Synovial fluid and chondrocytes

Synovial fluid (1-1.5 ml) was extracted from the affected site of 58 OA patients (32 cases of knee and 26 cases of hip) before the initiation of therapy. Collection of synovial fluid samples was performed using a syringe under local anesthesia. Synovial fluid (1-1.5 ml) was also extracted from the knee of 32 healthy controls and the hip of 26 healthy controls to match OA group. All synovial fluid samples were immediately transferred to a liquid nitrogen sink for long-term storage.

Cell model used in this study were primary chondrocytes from OA patients (402OA-05A, Sigma-Aldrich, USA). Chondrocytes were cultivated with Chondrocyte Growth Medium (PromoCell) in a 5% CO_2_ incubator at 37 °C. Subculture was performed at a ratio of 1:8. Cells used in subsequent experiments were collected at passage 1–2. In cases of osteopontin treatment, osteopontin (Sigma-Aldrich) was used to treat chondrocytes at dosages of 0, 1, 5 and 10 µg/ml for 48 h before use.

### Cell transfections

Osteopontin and HOTAIR backbone expressing vectors were constructed with pcDNA3.1 vector (Invitrogen). Negative control (NC) siRNA and HOTAIR siRNA were also purchased from Invitrogen. Vector (1 µg) or siRNA (40 nM) was transfected into 10^8^ chondrocytes using lipofectamine 2000 (Invitrogen). Empty vector or NC miRNA transfection was performed to serve as NC, and control (C) cells were untransfected cells. Subsequent experiments were performed 48 h later.

### ELISA

Levels of osteopontin in cell culture medium were measured using Osteopontin Human ELISA Kit (Catalog # BMS2066, Invitrogen). The analytical sensitivity was 0.26 ng/mL, and assay range was 0.47-30 ng/mL.

### RNA preparations

Isolation of RNA from both synovial fluid and chondrocytes was performed using RNAzol reagent (Invitrogen), followed by genomic DNA removal performed by incubating all RNA samples with DNase I for 2 h at 37 °C. To determine RNA integrity, RNAs were separated using 5% Urine-PAGE gel. To determine RNA purity, OD 260 and 280 ratios were measured, and 260/280 ratio was calculated.

### RT-qPCR

RNA samples with a 260/280 ratio close to 2.0 were revere transcribed into cDNA samples using ReverTra Ace™ qPCR RT Master Mix (TOYOBO). With cDNA samples as template, SYBR Premix Ex TaqTM II (Takara, Japan) was used to perform qPCRs to measure the mRNA levels of osteopontin and HOTAIR expression. GAPDH was used as internal control of all qPCRs. Three technical replicates were included in each experiment, and 2^−ΔΔCt^ method was used to normalize mRNA expression levels of osteopontin and HOTAIR to GAPDH. Primer sequences were: forward, 5’-GGAAAGATCCAAATGGGACCA-3’ and reverse, 5’-CTAGGAATCAGCACGAAGCAAA-3’ for HOTAIR; forward, 5’-GTCTCCTCTGACTTCAACAGC-3’ and reverse, 5’-ACCACCCTGTTGCTGTAGCCA-3’ for GAPDH; forward, 5’-GCAGTCATCCTTCTCTCAGT-3’ and reverse, 5’-GTATGCAGTAGCTTGTTACTT-3’ for GAPDH.

### Cell Counting Kit 8 (CCK-8) assay

Proliferation of chondrocytes in different transfection groups was analyzed by performing CCK-8 assay using a kit (ab228554, Abcam). Chondrocytes were transferred to a 96-well plate (5000 cells in 0.1 ml per well) and cells were cultivated at 37 °C. CCK-8 solution was added to reach 10% at 1.5 h before the measurement of OD values at 450 nm, which was performed every 24 h for a total of 4 days.

### Statistical analysis

PCRs were performed in three technical replicates, and all other experiments were performed in three independent biological replicates. Mean ± SD values were calculated. The difference between OA and control groups was explored by unpaired t test. One-way ANOVA and Tukey’s test were used to explore differences among multiple groups. Linear regression was used to compare multiple groups. Correlations were explored by linear regression. *p* < 0.05 was considered statistically significant.

## Results

### HOTAIR and osteopontin were upregulated in OA

**Fig. 1:**
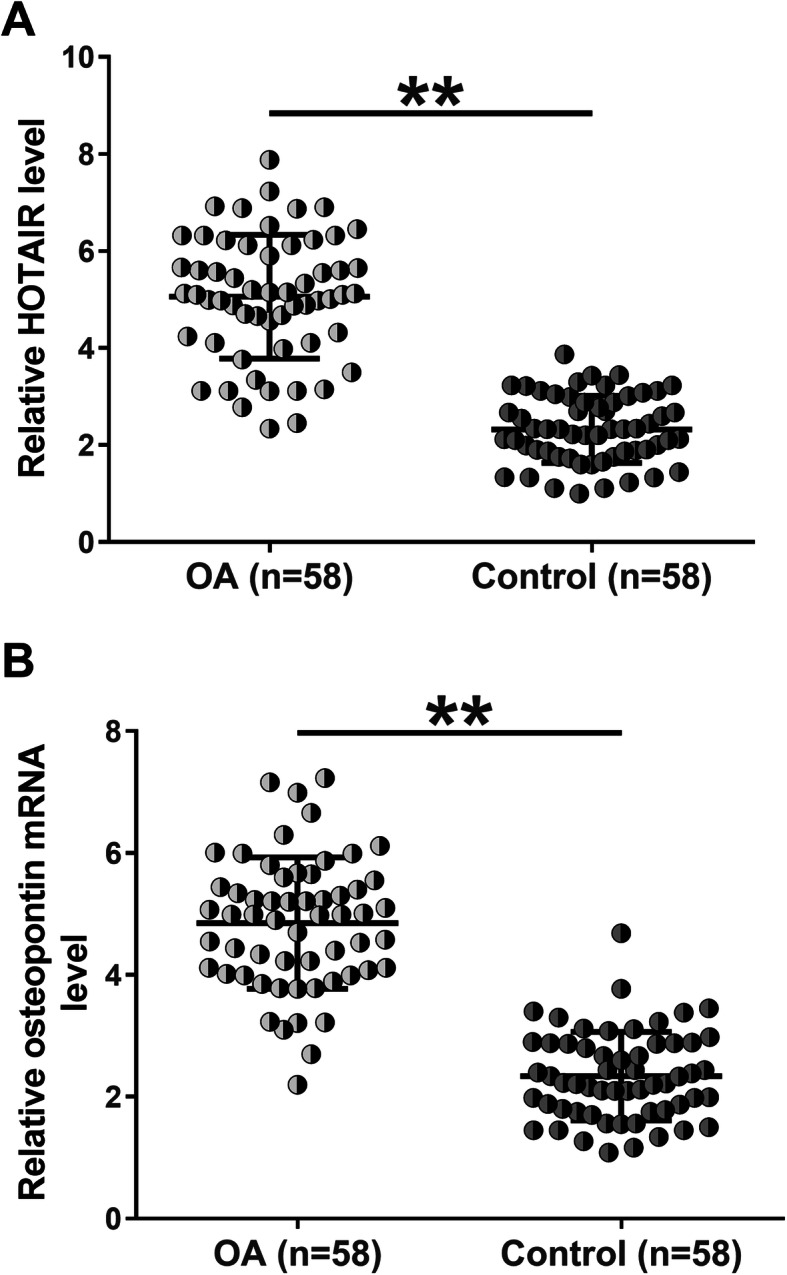
HOTAIR and osteopontin were upregulated in OA. Synovial fluid isolated from both OA patients (*n* = 58) and healthy controls (*n* = 58) was subjected to RNA isolation and RT-qPCR to measure the mRNA expression of HOTAIR and osteopontin. qPCRs were performed in triplicate and mean values were presented. **,*p* < 0.01

Synovial fluid samples isolated from both OA patients (*n* = 58) and healthy controls (*n* = 58) were subjected to RNA isolation and RT-qPCR to measure the mRNA expression levels of HOTAIR and osteopontin. Compared with Control group, the mRNA expression of HOTAIR(Fig. 1a, *p* < 0.01) and osteopontin (Fig. 1b, *p* < 0.01) was significantly upregulated in OA group. Therefore, our data suggested that upregulated expression of HOTAIR and osteopontin may involve in OA.

### HOTAIR and osteopontin were positively correlated across OA patients

**Fig. 2:**
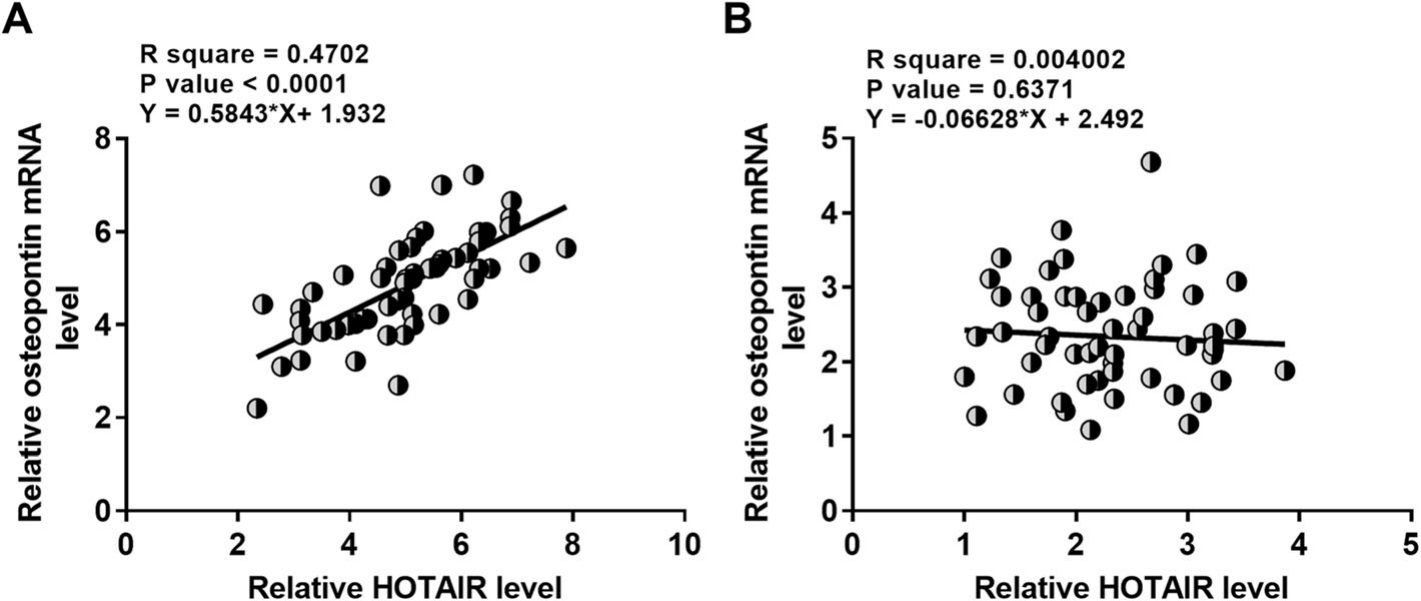
HOTAIR and osteopontin were positively correlated across OA patients. Correlations between HOTAIR and osteopontin mRNA across synovial fluid samples from both OA patients (**a**) and healthy controls (**b**) were analyzed by performing linear regression analysis

Correlation between HOTAIR and osteopontin mRNA across synovial fluid samples from both OA patients (Fig. 2a) and healthy controls (Fig. 2b) was analyzed by performing linear regression analysis. The results revealed a significant and positive correlation between HOTAIR and osteopontin mRNA across synovial fluid samples from OA patients, but not from healthy controls.

### Osteopontin induced the upregulation of HOTAIR expression in chondrocytes from OA patients

**Fig. 3:**
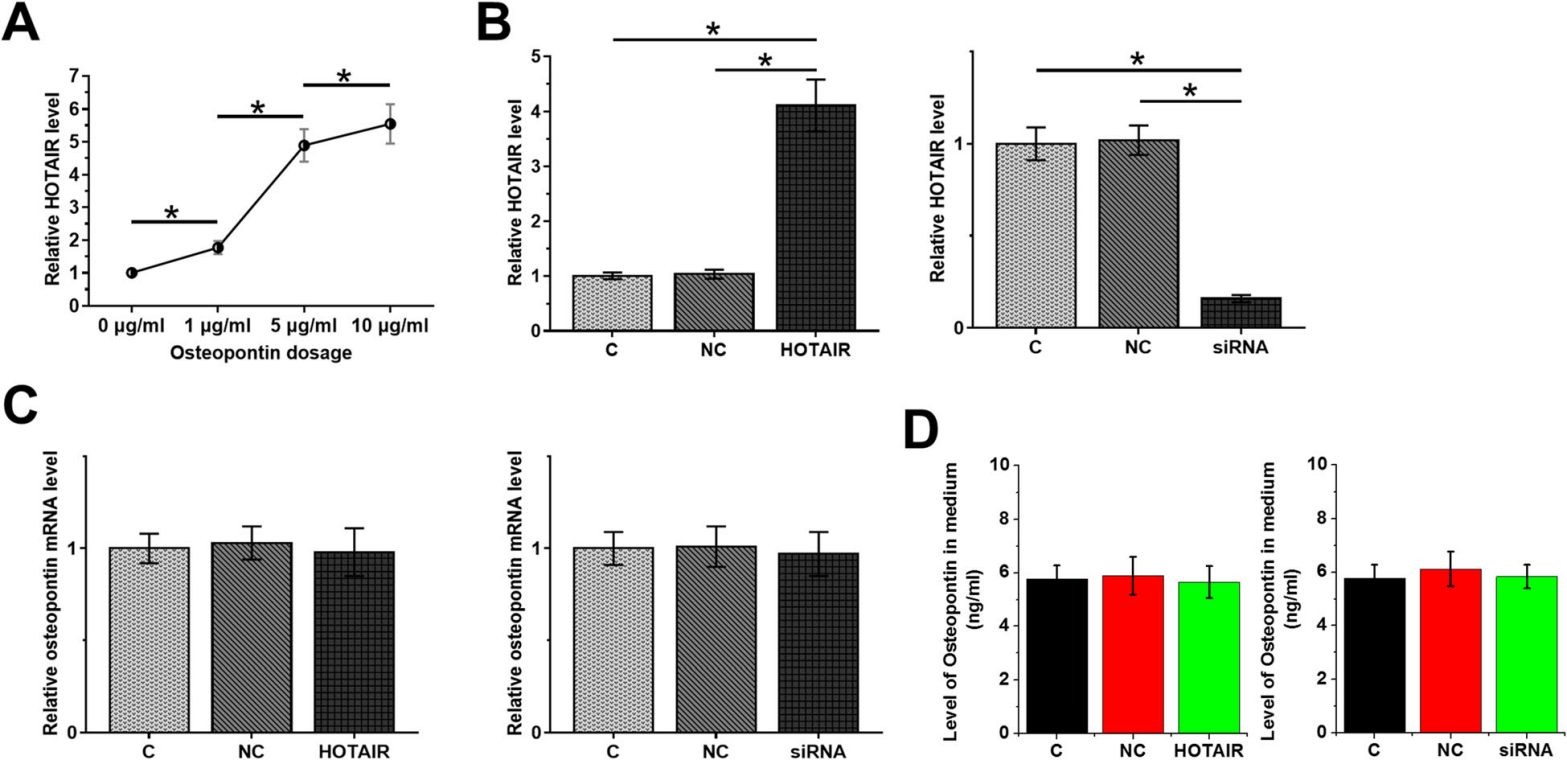
Osteopontin induced the upregulation of HOTAIR in chondrocytes from OA patients. To explore the interaction between them, osteopontin was used to treat chondrocytes at dosages of 0, 1, 5 and 10 µg/ml, followed by measuring the expression of HOTAIR by RT-qPCR (**a**). To test whether HOTAIR can also regulate osteopontin, HOTAIR overexpression and knockdown was achieved by transfecting HOTAIR expression vector or siRNA into chondrocytes, followed by confirmation of transfections by RT-qPCR (**b**). The effects of HOTAIR overexpression and knockdown on the expression of osteopontin were analyzed by RT-qPCR (**c**) and ELISA (**d**). All experiments were repeated 3 times and mean ± SD values

The significant correlation between HOTAIR and osteopontin in OA patients suggested the potential interaction between them in OA. To explore the interaction between them, osteopontin was used to treat chondrocytes at dosages of 0, 1, 5 and 10 µg, followed by measuring expression of HOTAIR by RT-qPCR. It was observed that osteopontin treatment upregulated HOTAIR expression in a dose-dependent manner (Fig. 3a, *p* < 0.05). To test whether HOTAIR can also regulate osteopontin, HOTAIR overexpression and knockdown was achieved by transfecting HOTAIR expression vector or siRNA into chondrocytes, followed by confirmation of transfections by RT-qPCR (Fig. 3b, *p* < 0.05). Compared with C and NC groups, HOTAIR overexpression and knockdown failed to significantly affect the expression of osteopontin (Fig. 3c). ELISA data also showed that HOTAIR overexpression and knockdown failed to significantly affect the secretion of osteopontin from chondrocytes (Fig. 3d). Therefore, osteopontin is likely an upstream inducer of HOTAIR expression.

### Osteopontin promoted the proliferation of chondrocytes through HOTAIR

**Fig. 4:**
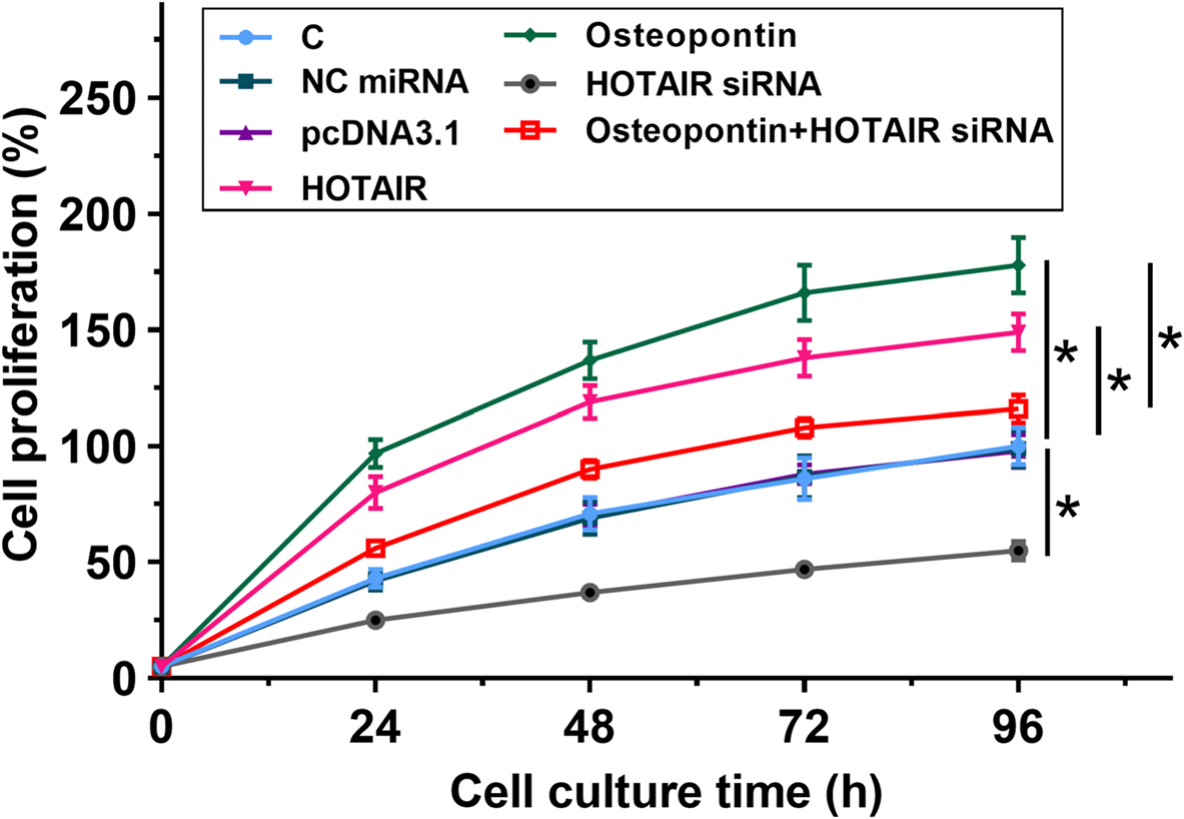
Osteopontin promoted the proliferation of chondrocytes through HOTAIR. The role of osteopontin and HOTAIR overexpression and HOTAIR knockdown in regulating the proliferation of chondrocytes was analyzed by performing CCK-8 assay. All experiments were repeated 3 times and mean ± SD values

The role of osteopontin and HOTAIR overexpression and knockdown in regulating the proliferation of chondrocytes was analyzed by performing CCK-8 assay. It was observed that osteopontin and HOTAIR overexpression promoted the proliferation of chondrocytes, while HOTAIRE knockdown inhibited the proliferation of chondrocytes. In addition, HOTAIR knockdown reduced the effects of osteopontin treatment on cell proliferation (Fig. 4, *p* < 0.05).

## Discussion

This study explored the interactions between osteopontin and HOTAIR in OA. We found that osteopontin and HOTAIR were both upregulated in OA. Moreover, osteopontin might induce the expression of HOTAIR to promote the proliferation of chondrocytes derived from OA patients.

HOTAIR is a well-established critical player with oncogenic roles in cancer biology [14]. Several recent studies also showed that HOTAIR has multiple functions in the pathology of OA. HOTAIR is overexpressed in OA and promotes IL-1β-induced MMP overexpression [15]. Overexpression of HOTIAR in OA interacts with miR-17-5p/FUT2/β-catenin axis to promote disease progression [16]. In another study, HOTAIR was reported to interact with lncRNA PACER to participate in the regulation of chondrocyte apoptosis[17]. Consistently, our results were in consistent with previous study that the expression of HOTAIR was upregulated in OA.

Although the involvement of HOTAIR in OA has been reported by several previous studies, the interaction of HOTAIR with osteopontin, a critical player in OA [10, 11], is still known. Osteopontin was reported to regulate HOTAIR in cancer cells [13]. In our study, we showed that HOTAIR was also upregulated by osteopontin in chondrocytes. However, HOTAIR overexpression and knockdown failed to regulate the expression of osteopontin. Therefore, our study characterized osteopontin as an upstream regulator of HOTAIR in OA.

Chondrocytes are the only mature cells in healthy cartilage, and reduce proliferation and increase apoptosis of chondrocytes contribute to the progression of OA [18]. Both our study and previous studies showed enhanced effects of osteopontin on the proliferation of chondrocytes [10, 11]. Therefore, osteopontin may play protective roles in OA by promoting the proliferation of chondrocytes. However, osteopontin is also a pro-inflammatory factor in OA and an inflammatory response contribute to the development of OA [10], suggesting that it may also promote the progression of OA by inducing inflammation. Therefore, the use of osteopontin as a target for the treatment of OA should be carefully evaluated. It has been well established that lncRNA expression was frequently altered in OA [19]. The altered expression of lncRNAs may participate in OA progression by regulating the proliferation of chondrocytes [20, 21]. For instance, lncRNA SNHG5 regulates the axis of miR-26a/SOX2 to increase the proliferation of chondrocytes [20]. In addition, lncRNA XIST regulates miR-211 to affect the proliferation of OA chondrocytes through CXCR4 and MAPK [21]. In this study we showed that HOTAIR could also regulate the proliferation of chondrocytes. However, the mechanism that mediates the function of HOTAIR remains to be explored.

However, this study was limited by the small sample size. Our future studies will enroll more OA patients and health controls to further confirm our conclusions. In addition, the in vivo interaction between osteopontin and HOTAIR in OA is unknown. Animal model experiments are needed to further explore their roles in OA. Besides that, levels of osteopontin in this study were only measured in cell culture, but not in synovial fluid. Although the levels of osteopontin in cell culture medium may also reflect the secretion of osteopontin under in vivo conditions, the level of osteopontin in synovial fluid should also be analyzed in future studies.

## Conclusions

In conclusion, osteopontin and HOTAIR are overexpressed in OA. Moreover, osteopontin induces the expression of HOTAIR to promote the proliferation of chondrocytes.

## Data Availability

The datasets generated and/or analyzed during the current study are not publicly available due research design but are available from the corresponding author on reasonable request.

## Abbreviations

OA: Osteoarthritis
lncRNAs: Long non-coding RNAs
C: Control
